# Why does strawberry fruit weight distribution show positive skewness? A simulation model reveals the underlying processes of fruit production

**DOI:** 10.3389/fpls.2023.1255724

**Published:** 2023-12-14

**Authors:** Zhihao Cao, Zhenfeng Jiang, Guanghui Liu, Yong Wang, Hongchun Qu

**Affiliations:** ^1^ College of Information Science and Engineering, Zaozhuang University, Zaozhuang, China; ^2^ Precision Agriculture Devision, Neumann (Shandong) Institute of Internet of things Co., Ltd, Zaozhuang, China; ^3^ Agricultural Machinary Department, Shandong Mingyuan Intelligent Equipment Co., Ltd, Zaozhuang, China

**Keywords:** fruit weight distribution, greenhouse strawberry, bee pollination, simulation modelling, log-normal distribution

## Abstract

It is widely accepted that the weight distribution of plant fruits conforms to a standard normal distribution. However, some overlooked evidence suggests that some fruits, including strawberries, exhibit positive skewness in fruit weight distribution. This intriguing observation has received limited attention thus far. To shed light on this phenomenon, we conducted a comprehensive simulation study utilizing greenhouse-grown strawberries as our research subject. We modeled the entire process from bee pollination to pollen fertilization on the stigma and fruit growth. The experimental results demonstrated the reliability of the proposed simulation model and revealed that the positive skewness of the fruit weight distribution was the result of the multiplication of several complex intermediate variable distributions, which led to an approximately lognormal distribution. The simulation model and the derived conclusions presented in this paper offer a plausible explanation for the weight distribution patterns observed in strawberry production systems. Furthermore, research results have the potential to be applied to other berry plants that undergo similar bee pollination processes, thereby expanding our understanding of fruit weight distributions across different species.

## 1 Introduction

Strawberries (*Fragaria × ananassa* Duch) are grown extensively worldwide and are mainly pollinated by bees (Abrol et al., 2019; Menzel, 2023). Fruit weight is affected by intrinsic and extrinsic factors such as genotype (Pereira et al., 2021), seed number within a berry, and environmental factors (Dai et al., 2011). Seed number is typically positively associated with fruit weight and is significantly influenced by pollination efficiency (Gray and Coombe, 2009). Precisely determining fruit weight is essential in horticulture for estimating yield and the proportion of marketable fruits (Pathak et al., 2016; Stanich et al., 2016). However, despite its significance, there is a dearth of published research concerning the fundamental distribution of strawberry fruit weights. Gaining a deeper understanding of this distribution and why it forms is valuable for expanding our knowledge regarding the interactions among various factors and processes in ecological contexts. The exploration of plant fruit weight distribution can provide valuable insights into the reproductive strategies implemented by plants and their adaptability within a particular environment. Moreover, by understanding the factors that influence the distribution of fruit weight, strategies aimed at optimizing strawberry cultivation practices (Cao et al., 2023) can be devised for large-scale production systems.

Some researchers have analyzed fruit weights by assuming that the weight follows a standard normal distribution. However, some overlooked evidence suggests that some fruit weights do not conform to a standard normal distribution and instead exhibit positive skewness, that is, with a higher proportion of small fruits than large fruits. For example, Menzel found that the strawberry marketable yield ranged from 48 to 890 g per plant, with a median of 321 g per plant and a positive skewness in weight distribution (Menzel, 2023). Chen et al. found that the distribution of single fruit weight in pears exhibited highly positive skewness characteristics (Chen et al., 2016). Although the fruit weight distribution ranged from 115 to 600 g, it was concentrated in the range of 200–300 g. Webb et al. discovered that the weight distribution of apples was slightly positively skewed since the distribution has been shown to fit a normal curve (Webb et al., 1980). Marini et al. studied the quality distribution of apple fruits in an orchard and found that the fruit weight was basically normally distributed (Marini et al., 2019). However, this distribution was slightly skewed to the right, with a skewness value of 0.948. The authors believed that the reason for the skewness was due to planting strategies. Naor et al. found that the quality distribution of nectarine fruit also exhibited positive skewness characteristics (Naor et al., 1999). Zhao et al. found that the overall quality distribution of loquat fruit followed a positively skewed normal distribution, with a skewness of 0.96 (Zhao et al., 2021). Medda et al. found that the weight per fruit of myrtle ranged from 0.15 to 0.50 g but was mainly concentrated in the range of 0.30 to 0.35 g, exhibiting positive skewness characteristics (Medda and Mulas, 2021). Searle et al. found that the weight distribution of buttercup squash exhibited an approximately normal distribution that was skewed towards small fruits (Searle et al., 2003). Pereira et al. analyzed tomato fruit weight and found that a high proportion of the fruit weight was in the medium to small range and that the proportion of high-quality fruit was relatively low (Pereira et al., 2021). The skewness in these distributions is relatively weak; thus, it has not aroused strong interest from the scientific community. The only interpretation (Marini et al., 2019) suggests that plants encounter constraints such as nutrient deficiency, pests, and diseases during their growth, leading to inhibited growth and a higher proportion of smaller fruits. However, other factors and their complex interactions during fruit production might also be significant contributors to this phenomenon.

To investigate the interesting phenomenon of positive skewness in fruit weight distribution, we used the Japanese strawberry cultivar *Beni hoppe* in a greenhouse as the research object because of its extensive history of study (Żebrowska, 1998; Hennessy et al., 2021). According to the central limit theorem, when a multitude of independent random variables are aggregated, their summation tends to approximate a normal distribution (Bertsekas and Tsitsiklis, 2008; D’Agostino, 2017). However, if one variable can be regarded as the multiplicative product of multiple independent factors, then this variable can be considered to have a lognormal distribution. In the realm of natural sciences, the lognormal distribution has frequently emerged as a compelling approximation (Mouri, 2013), characterized notably by its positive skewness. A lognormal process is the statistical realization of the multiplicative product of many independent random variables, each of which is positive. From an ecological standpoint, in the process of strawberry growth, from bee pollination to final fruit ripening, there are many complex intermediate processes, and pollination processes are driven by the accumulation of these intermediate processes. Thus, in this paper, the hypothesis that the positive skewness observed in the distribution of fruit weights arises from the underlying lognormal distribution, a consequence of the multiplication and accumulation of diverse and intricate intermediate variable distributions throughout the strawberry growth process, is proposed. These intermediate processes chiefly encompass the pivotal interplay of bee pollination, pollen fertilization, and fruit growth, entailing the participation of a myriad of independent pollen grains.

## 2 Materials and methods

From a botanical standpoint, the growth process of strawberry fruit can be divided into three independent stages: bee pollination, pollen fertilization, and fruit growth. In this paper, we specifically focused on investigating the growth of the Japanese cultivar *Beni hoppe* within a greenhouse environment. The journey of an individual strawberry pollen grain, originating from its production to the ultimate transformation into achenes on the fruit, encompasses a multifaceted process. The collective involvement of numerous pollen grains ultimately determines the distribution of strawberry fruit weight. In this paper, we developed a mathematical model to describe this process.

### 2.1 Bee pollination process

The initial stage of pollinating greenhouse strawberries involves the visitation of flowers by bees (Wietzke et al., 2018). *Beni hoppe* strawberry flowers typically bloom for a period of 5 days (McGregor, 1976). Under favorable conditions, bees venture out of their hives to forage daily and follow a specific pattern when visiting strawberry flowers (Skorupski et al., 2006; Opstad and Sonsteby, 2008). Drawing from data obtained from actual greenhouse experiments and our previous simulation model research (Cao et al., 2023), when the number of bees is basically equivalent to the number of strawberry plants in a greenhouse, the average frequency *λ* of bee visits to one open strawberry flower is approximately 4.5 times per day (Chagnon et al., 1989; Li et al., 2006), with each visit lasting between 6 and 10 seconds (Chen et al., 2011). It is important to note that the visit frequency *λ* is influenced by the bee density in the greenhouse during actual cultivation.

From this comprehensive view of the daily foraging process of bees, it can be observed that the visitation of a flower by a bee is a relatively rare occurrence, considering the gap between the overall time bees spend foraging and the duration of their visits to individual flowers. Although bees spend a considerable amount of time outside their hives foraging daily (approximately 7 hours), the probability of visiting a specific flower is low, and the duration of each visit is extremely short (approximately 6 seconds) (Li et al., 2006). Furthermore, each bee’s flower-visiting behavior is independent of one another. Research has shown that the age of the flower does not affect the bee’s visiting behavior, meaning that the probability of bees visiting strawberry flowers of different ages is statistically the same. Therefore, the number of times *X_n_* that flowers are visited by bees on the *n* ^th^ day (i.e., the age of the flower is *n* days) can be described using the Poisson distribution *X_n_* ~ *P*(*λ*), and *X*_1_, *X*_2_, *X*_3_, *X*_4_, and *X*_5_ all follow the same distribution pattern. The probability function of *X_n_* is shown in Equation 1. More validation processes are provided in the **Supplementary Materials**.


(1) $$P\left(X_{n}=k;n\right)=\frac{\lambda^{k}}{k!}e^{-\lambda }$$

The anthers situated on the stamens of strawberry flowers bear the pollen that is essential for pollinating the ovules. With each visit to a strawberry flower, a bee extracts pollen from the anthers and deposits a portion of it onto the stigma through its body. A honeybee deposits approximately 25 active pollen grains per visit (Chagnon et al., 1989). Hence, the number of pollen grains deposited on the stigma of a strawberry flower on the *n* ^th^ day, denoted as *Y_n_*, can be expressed using Equation 2. Notably, the number of pollen grains deposited on the stigma is directly proportional to the number of bee visits *X_n_*.


(2) $$Y_{n}=25*X_{n}$$

### 2.2 Pollen fertilization process

After the deposited pollen successfully falls on the stigma through bee foraging activity, the ovule fertilization process begins, which is comprised of two stages: the receiving stage and the accepting stage (Lata et al., 2018).

During the receiving stage, the probability of the stigma receiving pollen grains is positively correlated with stigma receptivity (Zhang et al., 2019). Stigma receptivity is primarily determined by the age of the flower, and its viability peaks within three days of blooming and sharply declines after five days (Vaissiere et al., 1996; Connelly et al., 2015). The possibility of the stigma of a flower receiving pollen grain on the *n* ^th^ day can be denoted as $P_{n}^{r}$ , as shown in Equation 3.


(3) $$P_{n}^{r}=e^{-0.01*n^{3.6}}$$

During the accepting stage, the pollination process is primarily influenced by the self-pollen compatibility of the strawberry cultivar. It is widely accepted that strawberry flowers are hermaphroditic and self-compatible to some extent but not entirely (Dinh Dung et al., 2022). Therefore, even if the stigma has high receptivity, it may not induce pollen tube growth. Relevant research has shown that the pollen self-compatibility probability of the *Beni Hoppe* cultivar is approximately 80% (Cao et al., 2023; Hsu and Lu., 2018).

Only when the pollen satisfies both the receiving and accepting stages can it combine with the ovules. In this model, the fertilization possibility of a flower on the *n* ^th^ day is denoted as $P_{n}^{f}$ , as shown in Equation 4.


(4) $$P_{n}^{f}=0.8*e^{-0.01*n^{3.6}}$$

On the *n* ^th^ day, the *Y_n_* pollen grains placed on the stigma have a fertilization probability of $P_{n}^{f}$ , and each successful fertilization event follows a binomial distribution. On the *n* ^th^ day, the number of pollen grains that successfully fertilized the flower follow a binomial distribution $Z_{n}∼B\left(Y_{n},P_{n}^{f}\right)$ . It can be roughly estimated that when *n* = 1, $Y_{1}\;*P_{1}^{f}\approx 158$ , which is a statistically large value, *Z_n_* is approximately a normal distribution. When *n*=5, the value of $P_{5}^{f}$ is extremely small, and $Y_{1}*P_{5}^{f}\approx 0.6$ ; as a result, statistically, *Z_n_* is approximately a Poisson distribution. Therefore, flower age is an important factor that affects the distribution of daily fertilized pollen grains.

Equation 5 illustrates that *Z_sum_*, the cumulative number of fertilized pollen grains over a period of five days, is the sum of *Z*_1,_ *Z*_2,_ *Z*_3,_ *Z*_4,_ and *Z*_5_. Upon successful fertilization of the ovule, each pollen grain matures into an achene within the flower’s receptacle. Achenes, resulting from fertilized ovules, are large and surrounded by well-developed fleshy tissue, whereas the achenes resulting from unfertilized ovules are less voluminous and closer together. Therefore, the number of achenes produced is equivalent to the number of pollen grains that have been successfully fertilized, denoted as *Z_sum_*.


(5) $$Z_{sum}=\sum_{i=1}^{5}Z_{i}$$

### 2.3 Fruit growth process

The development of the receptacle is significantly impacted by the achenes, which have the ability to secrete hormones that stimulate fruit growth. In practical cultivation, *Beni Hoppe* strawberries typically weigh approximately 14–15 g, and it is widely accepted that fruits must weigh over 10 g to be considered marketable (Cao et al., 2023). The weight of a berry is proportional to the number of fertilized ovules (achenes), and the number of stigmata per flower determines its potential weight (Abbott et al., 1970). Drawing from our proposed simulation model and previous research (Dung et al., 2021; Cao et al., 2023; Menzel, 2023), we determined that there exists a linear correlation between fruit weight *W* and the total number of achenes *Z_sum_*, which can be represented by Equation 6.


(6) $$W=0.05*Z_{sum}+2.0$$

However, there are significant residuals between the actual fruit weight and the linear fitting results that cannot be disregarded. During simulation modelling, accurately describing the residuals exhibited by fruit weight using only a fitting line is challenging, making it even more difficult to simulate a more realistic distribution of strawberry weights. To address this issue, we introduced a residual term *Bias* to the fitting equation to account for this deviation. In this paper, we assumed that *Bias* follows a normal distribution with a mean of 0, that is, *Bias* ~ *N*(0,*σ* ^2^). Assuming that the residual *Bias* follows a normal distribution is a common technique in machine learning and has been widely used in regression models (Maulud and Abdulazeez, 2020). More validation and computing processes are provided in the **Supplementary Materials**. The final fitting relationship for fruit weight *W* is shown in Equation 7.


(7) $$W=0.05*Z_{sum}+2.0+Bias$$

### 2.4 Statistical methods

In this study, we employed the one-sample Kolmogorov−Smirnov test to assess the distribution of the data. A p-value greater than 0.05 indicated that the data conform to a Poisson distribution. To evaluate if the differences between the simulated data and the empirical data are different, we applied the Kolmogorov−Smirnov test. A p-value exceeding 0.05 signified that there was no significant difference between the two sets of data. Moreover, a quantile−quantile (Q−Q) plot was used to verify the normality of the distribution.

We utilized SPSS 26 software for basic data analysis and hypothesis testing. Additionally, the stats package in Python (Virtanen et al., 2020) was employed for data fitting and analysis.

## 3 Experiments and results

Due to the complexity of fruit weight *W*, which is the result of the multiplicative product of intermediate variables *X_n_*, *Y_n_*, *Z_n_*, and *Z_sum_*, it is difficult to obtain the direct distribution function of *W* through conventional means. In this study, we employed Monte Carlo simulations to model and experiment with the growth process of strawberries.

The research comprised three experimental groups. The first group investigated the distribution of bee visitation and the associated pollen quantity during the intermediate stages. The second group deeply examined the distribution of fruit weight *W*. The third group conducted sensitivity analysis on crucial variable parameters during the intermediate stages, aiming to identify factors that could influence the distribution of fruit quality.

To mitigate the influence of randomness, we set the number of simulated flowers to 10,000. This larger sample size is expected to effectively reduce the impact of random variations in simulation.

### 3.1 Distribution of bee visitation and pollen quantity

**Figure 1:**
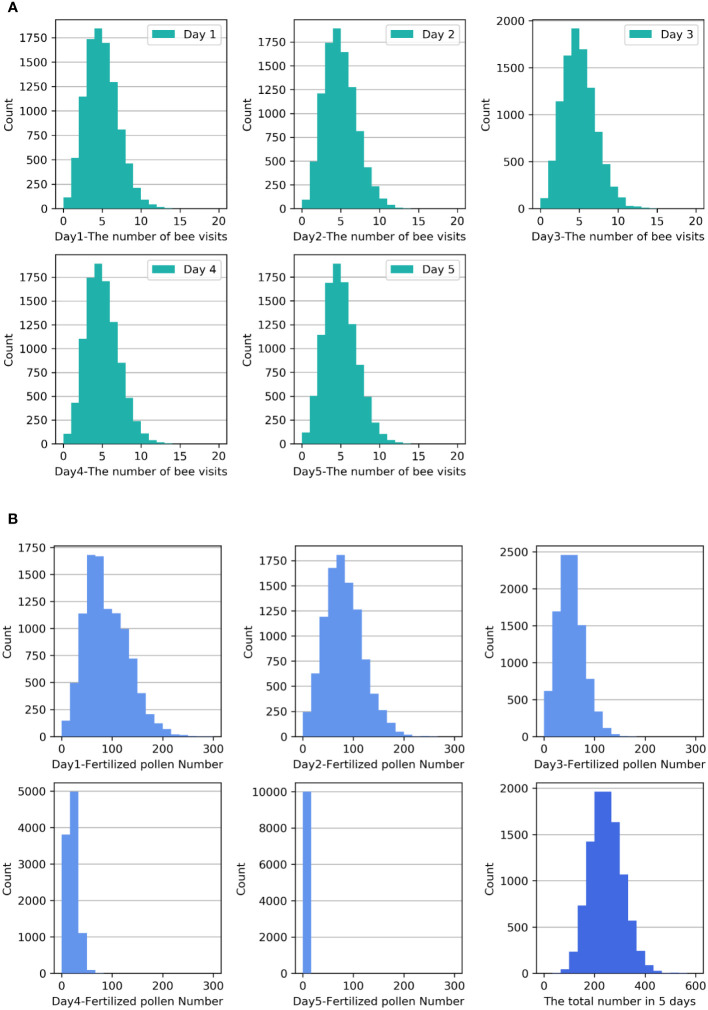
**(A)** Frequency histograms of the number of bee visits *X_n_* to one flower over the five-day period. **(B)** Frequency histograms of the pollen quantity *Zn* over the five-day period and the accumulated pollen quantity *Z_sum_* during the blooming period.

First, we investigated the distribution of bee visitation and the associated pollen quantity during the intermediate stages. In the simulation, it is assumed that the daily bee visit frequency *X_n_* of each flower follows a Poisson distribution with a parameter of 4.5 (Cao et al., 2023). The frequency histograms of *X_n_* are shown in **Figure 1A**. It can be observed that the frequency histograms are almost identical over the five days, and the probability of visit frequency of 4 is the highest. The frequency histograms of the pollen quantity *Y_n_* on the flower head over the five days are shown in **Figure 1B**. Since the pollen quantity *Y_n_* is directly controlled by the bee visit frequency *X_n_*, the histograms of the two variables are basically the same when the grouping number is the same.

The frequency histogram of the pollen quantity *Z_n_* that successfully fertilized the ovules over the five days is shown in the first five small diagrams in **Figure 1B**. From these diagrams, it can be observed that as the strawberry flowers age and the stigma activity decreases, the probability of successful pollen fertilization decreases significantly. The greatest probability of successful fertilization was observed when a pollen quantity of approximately 60 was distributed on the first two days, approximately 50 on the third day, and approximately 20 on the fourth day. Nevertheless, the probability of successful fertilization on the fifth day was exceedingly low. The frequency histogram in the last small diagram of **Figure 1B** displays the accumulated pollen quantity *Z_sum_* that successfully fertilized the ovules over the five-day period. The diagram indicates that the distribution of *Z_sum_* is positively skewed. Analysis reveals that the mean of *Z_sum_* is 245.70, with a skewness of 0.328 and a standard error of skewness of 0.024. The skewness Z score is 13.67, indicating moderate positive skewness.

### 3.2 Distribution of the fruit weight *W*

**Figure 2:**
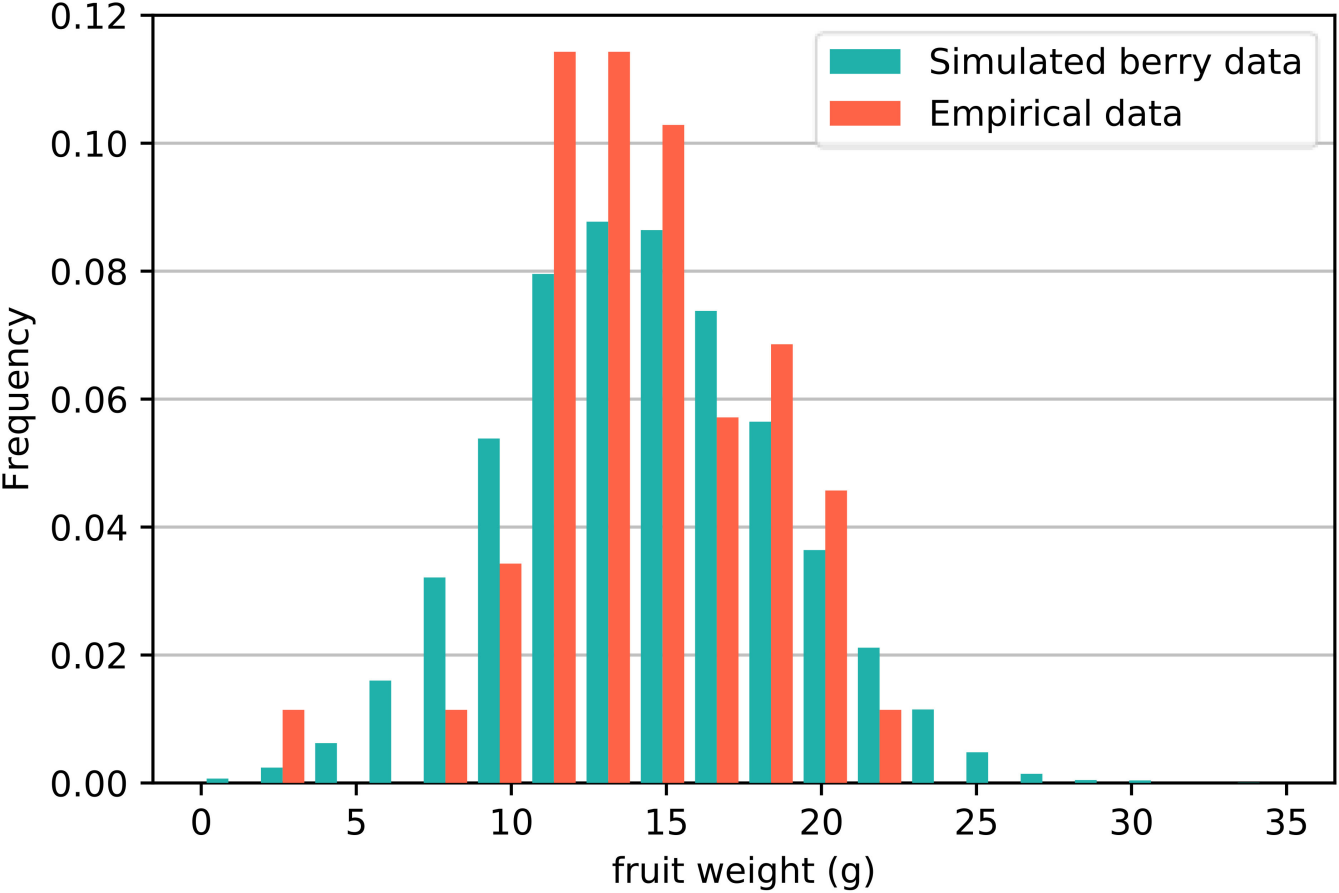
Distribution frequency histogram of strawberry fruit weight *W* obtained from simulations and empirical data. The Kolmogorov−Smirnov test revealed that there was no significant difference between the two sets of data.

Second, we investigated the distribution of fruit weight *W* with empirical data. **Figure 2** displays the distribution frequency histogram of strawberry fruit weight *W* obtained from the simulation results (green bar) and empirical data (orange bar). The diagram in **Figure 2** indicates that the frequency of fruit weight is highest at approximately 15 g with a maximum of approximately 28 g, which is consistent with the actual planting data. Analysis of the simulation results revealed that the mean of *W* is 14.6, with a standard deviation of 4.42 and a skewness of 0.108. The standard error of skewness is 0.024, and the skewness Z score is 4.5, indicating slight positive skewness. We employed the Kolmogorov−Smirnov test to evaluate the difference between the simulated data and the empirical data. The test results gave a p-value exceeding 0.05 (P = 0.862). Based on this outcome, it can be concluded that there is no significant difference in the weights of the fruits between the two sets of data.

**Figure 3:**
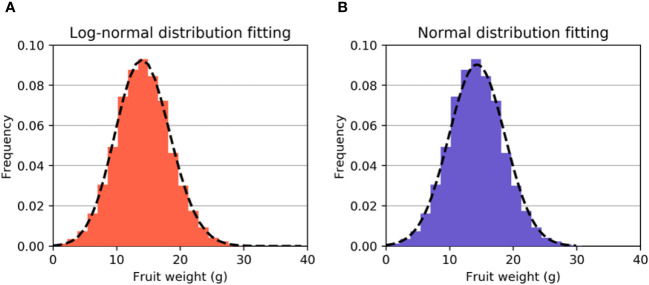
**(A)** Fitted curve of the lognormal distribution. **(B)** The fitted curve of the normal distribution. It can be observed that the fitting effect of the lognormal distribution is better.

As previously stated, we proposed a hypothesis that the ultimate weight of the fruit, denoted as *W*, follows a lognormal distribution as a result of the multiplicative intermediate processes. To fit the fruit weight *W* with the simulation results, we utilized the stats package. The fitting equation comprises three parameters: *s*, *scale*, and *loc*. The resulting probability density function obtained from the fitting is shown in Equation 8:


(8) $$f\left(x\right)=\left(\frac{1}{sy\sqrt{2\pi }}e^{-\;\frac{log^{2}\left(y\right)}{2s^{2}}}\right)/scale$$

where *y* = (*x* – *loc*)/*scale*. These results gave the parameters s = 0.0411, scale = 105.2339, and loc= -91.0223. **Figure 3A** displays the simulation experimental results for the lognormal distribution of fruit weight. The Kolmogorov−Smirnov test was employed to validate the hypothesis that the fruit weight *W* conforms to a lognormal distribution with a result of P = 0.91, which is considerably higher than 0.05. Hence, we can reasonably infer that our proposed hypothesis is valid.

As the data exhibited skewness, we conducted another fitting experiment using a normal distribution. The Kolmogorov−Smirnov test was performed to verify the normal distribution, with a result of p = 0.30, which is still higher than 0.05. The mean and standard deviation were computed to be 14.3 and 4.42, respectively. The experimental results suggest that the distribution of *W* can also be approximated by a normal distribution *N*(14.3,4.42^2^), as depicted by the fitted curve in **Figure 3B**. It can be observed that although the distribution of *W* can be modelled as either a lognormal or normal distribution, the fitting result of the lognormal distribution is evidently superior to that of the normal distribution.

**Figure 4:**
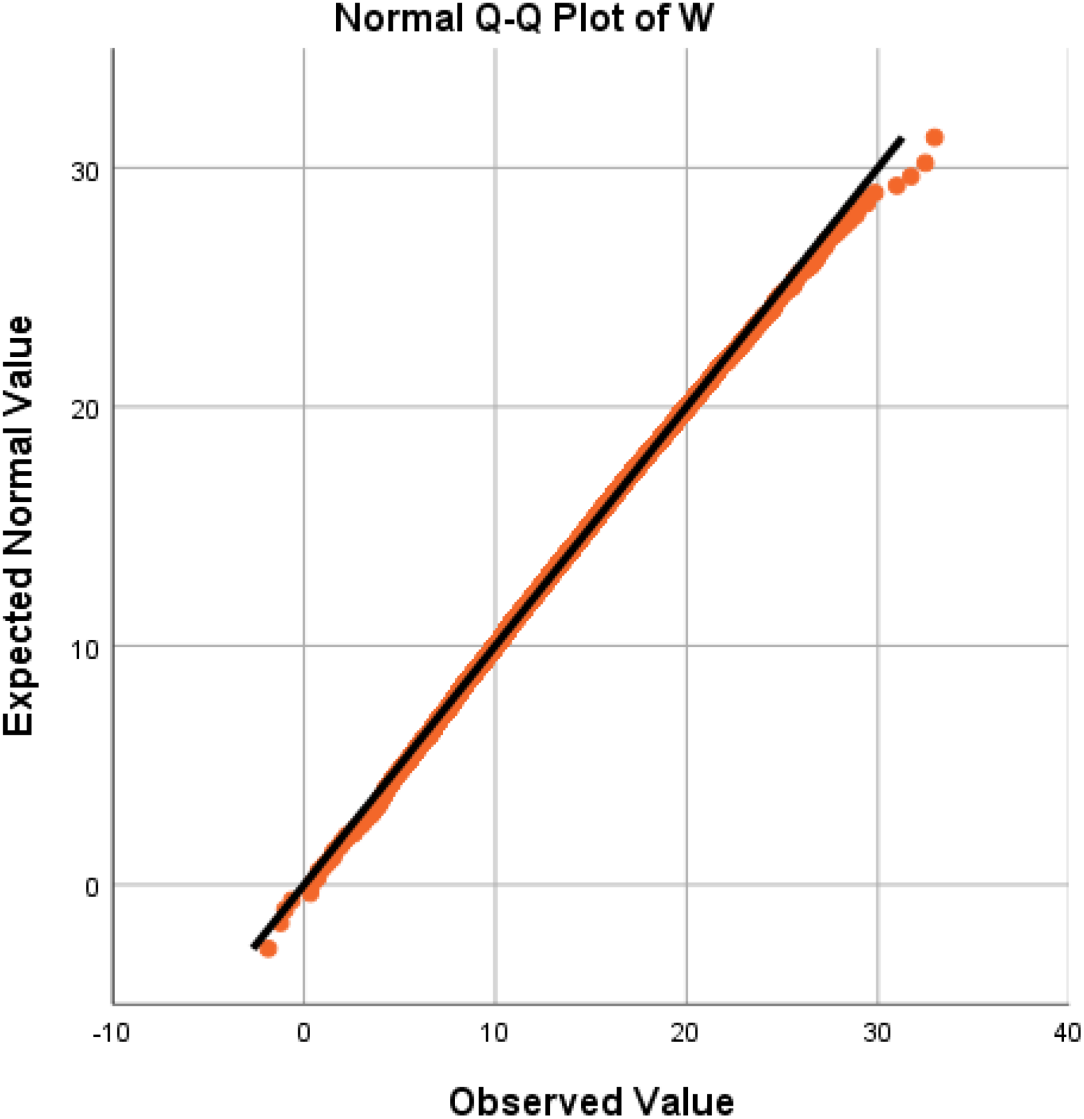
Verification of the normal distribution using a Q−Q plot.


We employed a quantile−quantile (Q−Q) plot to verify the normality of the *W* distribution once again, and the experimental results are depicted in **Figure 4**. The Q−Q plot reveals that the data points (orange dots) are closely distributed around the straight line, indicating robust normality of the fruit weight distribution. Consequently, the distribution *W* of strawberry fruit weight can be deemed a normal distribution with slight positive skewness. Given the relatively small skewness value, *W* essentially conforms to the normal distribution of *N* (14.3,4.42^2^) when disregarding the influence of positive skewness. It is commonly accepted that only strawberry fruits weighing over 10 g are marketable, and this proportion can be estimated to be approximately 83.40%, which is consistent with the actual planting results.

$$P\left(W>10\right)=1-\;P\left(\frac{W-14.3}{4.42}\leq \;\frac{10-14.3}{4.42}\right)=\phi \left(0.97\right)=\;83.4\%$$

### 3.3 Sensitivity analysis

The distribution of final fruit weight *W* is a result of multiple intermediate processes. Ultimately, a lognormal distribution is observed. In this section, we conducted sensitivity analysis to investigate the impact of three important variable parameters in the intermediate processes on fruit quality, including average fruit weight, marketable fruit ratio, and distribution skewness. Sensitivity analysis is widely utilized in machine learning (Baghban et al., 2019) and simulation (Kleijnen, 2010) to examine and predict the influence of input variables by allowing them to vary within their respective ranges. This method enables the study of how these variables impact the output of the model.

The first parameter to investigate is the average daily visitation number *λ*, which represents the density of bees and their visiting behavior towards plant flowers. The second parameter is self-pollen compatibility, which varies among different plant cultivars. Analyzing this parameter helps enhance model transferability. The third parameter is the fitting equation between seed quantity and fruit weight, which exhibits significant variations across different plants. Analyzing this equation allows for a deeper understanding of the positively skewed distribution, particularly across different species. By comprehending the factors that influence the distribution of strawberry fruit weight, we can identify the most significant factors contributing to variations in fruit quality. This enhances the interpretability of the model and increases its potential for application to other berry plants that undergo similar bee pollination processes.

We embarked on a sensitivity analysis of the average daily visitation of one flower by bees, which was denoted by *λ*. The value of *λ*, serving as the parameter of the Poisson distribution *X_n_* ~ *P*(*λ*), is contingent upon the bee density in the greenhouse and visiting behavior towards the flowers. In our simulation experiment, we set *λ* to 2.5, 3.0, 3.5, 4.0, 4.5, 5.0, 5.5 and 6.0. Then, we observed the average fruit weight, marketable fruit ratio, and the skewness of *W* distribution in the simulation results.

**Figure 5:**
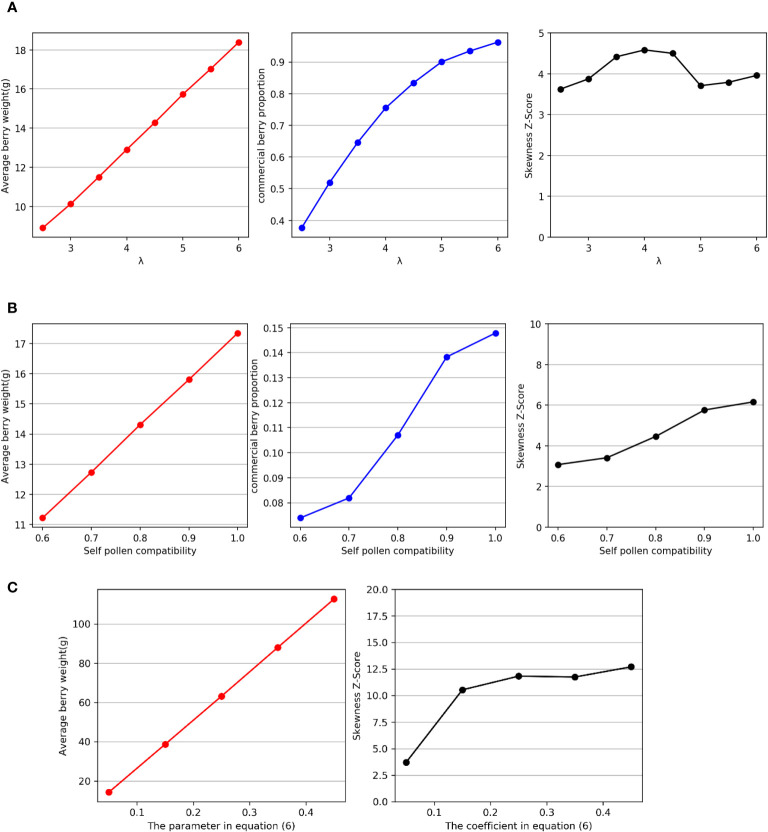
**(A)** Relationships between *λ* and average fruit weight, marketable fruit ratio, and distribution skewness. **(B)** Relationships between self-pollen compatibility and average fruit weight, marketable fruit ratio, and distribution skewness. **(C)** Relationships between the coefficient of *Z_sum_* in Equation 6 and the average fruit weight and distribution skewness.

As depicted in **Figure 5A**, the findings evince a positive linear correlation between *λ* and the average fruit weight, as well as a positive correlation between *λ* and the marketable fruit ratio. Nevertheless, the skewness Z score remains unaffected by *λ*, and in each scenario, the distribution of *W* manifests positive skewness.

Subsequently, we investigated self-pollen compatibility. Not only does self-pollen compatibility vary among different plants, but it also differs among different strawberry cultivars. Self-pollen compatibility is a crucial factor that affects pollen fertilization and is of great significance for plant genetic diversity. In this experiment, we adjusted this value in the simulation, setting it to 0.5, 0.6, 0.7, 0.8, 0.9, and 1.0, and observed the average fruit weight, marketable fruit ratio, and skewness of the *W* distribution.

As shown in **Figure 5B**, the results indicate a positive linear correlation between self-pollen compatibility and average fruit weight, as well as a positive correlation between self-pollen compatibility and marketable fruit ratio. Moreover, the experimental results also reveal that the degree of skewness in the distribution increases with increasing self-pollen compatibility. However, from the perspective of the change in skewness Z score, the impact of self-pollen compatibility on skewness is relatively weak.

Finally, we conducted a sensitivity analysis on the relationship between seed quantity and fruit weight by adjusting the coefficient of *Z_sum_* when fitting Equation 6. This coefficient approximates the proportion of fruit quality to seed quantity. It is worth noting that the seed number and weight of strawberry fruits differ from those of other fruits, such as apples (Webb et al., 1980; Raine et al., 2006; Marini et al., 2019), which results in a significant variation in this parameter among different crops.

We adjusted the value of this parameter to observe changes in the average fruit weight and distribution skewness. In the simulation experiments, we set this parameter to 0.05, 0.15, 0.25, 0.35, and 0.45. The experimental results, as shown in **Figure 5C**, indicate that the degree of skewness in *W* increases with the increase in the proportion of fruit weight to seed quantity, and significant differences can be observed.

## 4 Discussion

The results of the simulation experiments supported our hypothesis that the positively skewed distribution of strawberry fruit weight *W* can be attributed to the multiplicative effects of various complex distributions resulting from intermediate processes such as bee pollination, pollen fertilization, and fruit growth. Our research rectified the misconception that plant growth is influenced by factors such as nutrient deficiency, pests, and other environmental conditions, resulting in a higher proportion of smaller fruits and a positively skewed distribution of fruit weights.

These intermediate processes collectively lead to the manifestation of a lognormal distribution. By examining the intermediate processes of the model, we believe that the normal distribution characteristics of fruit weight *W* stem from the fact that the weight of the fruit is a result of the contributions of numerous seeds, and the probability of each seed being fertilized by pollen is independently and identically distributed.

The sensitivity analysis experimental results reveal that average daily visitation *λ* has no effect on the skewness of the distribution of fruit weight *W* but has a significant impact on the average fruit weight and the marketable fruit ratio. In addition to bee density, *λ* is also influenced by bee visiting behavior towards plant flowers. Moreover, the frequency of bee visits varies for each type of flower and is influenced by factors such as scent and nectar availability (Laverty, 1994). Therefore, we believe that these physiological factors of flowers are not the cause of the skewed distribution of fruit weight. This result also indicates that bee visitation behavior is an important factor affecting the weight of strawberry fruits, and it is crucial for strawberry growers to ensure an adequate number of bees in the greenhouse (Qu et al., 2017). The degree of skewness of the distribution of fruit weight *W* increases with increasing self-pollen compatibility. Based on the observed numerical fluctuations, it appears that the extent of the effect of self-pollen compatibility is limited and cannot fully explain the variations in skewness of fruit weight distribution among different plants. Therefore, we believe that although self-pollen compatibility does have a significant impact on fruit weight, it does not have a significant influence on the skewness of the distribution. The results from the third experimental group indeed indicate that there is a positive relationship between the proportion of fruit weight to seed quantity and the skewness of the weight distribution. Furthermore, based on the observed numerical fluctuations, this effect is more significant. Relevant data suggest that apple fruit weight distribution exhibits high skewness (Webb et al., 1980; Marini et al., 2019), with apple weight surpassing that of strawberries, while the seed quantity is significantly lower in apples compared to strawberries (Raine et al., 2006). From a pollination perspective, we can conclude that this is one of the reasons for the higher skewness in the distribution of apple fruit weight compared to strawberries.

The proposed model exhibits high reliability and transferability. We validated and refined the model using empirical data to model the bee pollination process and the relationship between fruit weight and seed quantity. The final average weight and distribution of strawberry fruits in the simulation were consistent with actual planting experience, indicating the reliability of the proposed model. Moreover, the model has the potential to be applied to other berry plants that undergo similar bee pollination processes (Qu and Drummond, 2018). On one hand, while the time that bees spend on different plant flowers may vary, the average number of daily visits typically conforms to the requirements of an approximate Poisson distribution (Malagodi-Braga and Kleinert, 2004; Woodcock et al., 2013). On the other hand, for many crops, although the number of seeds and fruit weight differ significantly from strawberries, the relationship between seeds and fruit weight can be fitted to a linear equation (Buccheri and Di Vaio, 2005; Raine et al., 2006; Gray and Coombe, 2009; Dai et al., 2011). Therefore, we believe that the model can be fine-tuned and applied to other crops.

However, the current model still has certain limitations. The growth of strawberry fruits in the simulated environment is idealized but neglects the effects of genotype and growth environment factors. In an attempt to incorporate these crucial factors into the model, we introduced the *Bias* variable in Equation 7. This addition aimed to simulate the inherent randomness associated with genotype and growth environment, recognizing their potential influence on fruit weight. However, we must recognize that this simplified approach may not fully capture the intricate relationships and complexities inherent in real-world scenarios. Our future research focus encompasses two aspects: incorporating genotype and growth environment factors into the model and expanding the applicability of this model to other berry plants that undergo similar underground pollination processes, such as apples and blueberries.

## 5 Conclusion

This study delves into an intriguing and often overlooked phenomenon in agricultural cultivation. To study this phenomenon, we proposed a mathematical model with Monte Carlo simulations to analyze the entire process of greenhouse strawberry pollination and fruit growth using the Japanese cultivar *Beni hoppe* as the research subject.

The proposed model demonstrates that the multiplication and accumulation of complex intermediate variable distributions during the growth process result in strawberry fruit weight following a lognormal distribution. This lognormal distribution is a significant factor that contributes to the positive skewness observed in the fruit weight distribution. The model and simulation results presented in this paper offer a reasonable explanation for the positively skewed weight distribution of strawberries, and this model has the potential to be applied to other bee-pollinated berry plants.

## Data availability statement

The original contributions presented in the study are included in the article/**Supplementary Material**. Further inquiries can be directed to the corresponding author.

## Author contributions

ZC: Investigation, Methodology, Formal Analysis, Writing – original draft. ZJ: Validation, Writing – original draft, Writing – review & editing, Visualization. GL: Data curation, Resources, Writing – original draft, Writing – review & editing. YW: Validation, Writing – original draft, Writing – review & editing, Visualization. HQ: Conceptualization, Formal Analysis, Funding acquisition, Methodology, Project administration, Supervision, Validation, Writing – original draft, Writing – review & editing.
