# Supplementary material for: Why does strawberry fruit weight distribution show positive skewness? A simulation model reveals the underlying processes of fruit production
Source: Front Plant Sci. 2023 Dec 14;14:1255724. doi: 10.3389/fpls.2023.1255724 (PMC11790150; doi:10.3389/fpls.2023.1255724)
Supplement: Supplementary file 1 [file DataSheet_1.pdf]

Codes in Python

```
# -*- coding: utf-8 -*-
"""
Created on Wed May 24 16:47:07 2023

@author: 10364
"""

import numpy as np
import random
import matplotlib.pyplot as plt
import math

flower_number = 10000

# Empirical data
weight_r = [18.61663660174187,
            17.529841524700522,
            19.2090663435753,
            11.60803203488377,
            13.739433478769461,
            15.247371840566046,
            13.435549687866466,
            12.287024807896508,
            14.822286576042726,
            18.626340768530177,
            15.40531471886315,
            15.745046353951276,
```

15.111349260594167,

13.2040768365293,

20.62853861361711,

17.15509954919837,

16.60971187792467,

8.874712807017405,

7.847393586706856,

12.212167881969593,

11.742910918133191,

15.457877117047682,

20.897631670192045,

17.99666391325702,

16.333958902907803,

12.280364529031928,

11.061197413325296,

15.40838438583645,

1.8196162502990365,

11.321874919918459,

18.39080838409134,

10.089466883230221,

12.735373604278811,

20.98340559685257,

11.378159642339789,

9.882598851204136,

21.587823906728385,

13.874486490283841,

10.85445720275445,

10.999525537391593,

12.220566276152692,

```
    13.511867207686944,
    13.39373182657575,
    14.065040140248723,
    14.342206098274596,
    16.875961740697946,
    19.4128578885391,
    15.810815007051037,
    13.912162815726267,
    11.813992136893111]

rou = 4.5

Visit_D1 = np.random.poisson(rou, flower_number)
Visit_D2 = np.random.poisson(rou, flower_number)
Visit_D3 = np.random.poisson(rou, flower_number)
Visit_D4 = np.random.poisson(rou, flower_number)
Visit_D5 = np.random.poisson(rou, flower_number)


All_Pollen_D1 = 25*Visit_D1
All_Pollen_D2 = 25*Visit_D2
All_Pollen_D3 = 25*Visit_D3
All_Pollen_D4 = 25*Visit_D4
All_Pollen_D5 = 25*Visit_D5

Compatible_Probability = 0.8

p = [0.99, 0.88, 0.59, 0.23, 0.04]
```

```
Receive_P1 = p[0]*Compatible_Probability
Receive_P2 = p[1]*Compatible_Probability
Receive_P3 = p[2]*Compatible_Probability
Receive_P4 = p[3]*Compatible_Probability
Receive_P5 = p[4]*Compatible_Probability

#Day 1
# Temp store the number of fertilized pollen
F_Pollen_D1 = [0]*flower_number
for n in range(flower_number):
    for i in range(All_Pollen_D1[n]):
        p = random.random()
        if p <= Receive_P1:
            F_Pollen_D1[n] = F_Pollen_D1[n] + 1
#Day 2
F_Pollen_D2 = [0]*flower_number
for n in range(flower_number):
    for i in range(All_Pollen_D2[n]):
        p = random.random()
        if p <= Receive_P2:
            F_Pollen_D2[n] = F_Pollen_D2[n] + 1

#Day 3
F_Pollen_D3 = [0]*flower_number
for n in range(flower_number):
    for i in range(All_Pollen_D3[n]):
        p = random.random()
        if p <= Receive_P3:
            F_Pollen_D3[n] = F_Pollen_D3[n] + 1
```

```
#Day 4
F_Pollen_D4 = [0]*flower_number
for n in range(flower_number):
    for i in range(All_Pollen_D4[n]):
        p = random.random()
        if p <= Receive_P4:
            F_Pollen_D4[n] = F_Pollen_D4[n] + 1

#Day 5
F_Pollen_D5 = [0]*flower_number
for n in range(flower_number):
    for i in range(All_Pollen_D5[n]):
        p = random.random()
        if p < Receive_P5:
            F_Pollen_D5[n] = F_Pollen_D5[n] + 1

F_Pollen_All = [0]*flower_number
for n in range(flower_number):
    F_Pollen_All[n] = F_Pollen_D1[n] + F_Pollen_D2[n] + F_Pollen_D3[n] + F_Pollen_D4[n]
+ F_Pollen_D5[n]



bias = np.random.normal(loc = 0 , scale= 3.0,size = flower_number)
weight = [0]*flower_number
for n in range(flower_number):
    weight[n] = F_Pollen_All[n]*0.05 + 2.0 + bias[n]

# weight calculation for blueberry
```

```
weight_b = [0]*flower_number
for n in range(flower_number):
    weight_b[n] = 0.0988 + math.log(F_Pollen_All[n])*0.31935


# Figure 1 蜜蜂访问次数分布
plt.subplots(figsize=(9, 6),dpi= 300)
plt.subplot(2,3,1)
plt.xlabel("Day1-The number of bee visits")
plt.ylabel("Count")
plt.grid(axis = "y",zorder = 0)
plt.hist(Visit_D1 , bins = 20, density = False, range = (0, 20),zorder = 10,color="lightseagreen",label="Day 1")
plt.legend()

plt.subplot(2,3,2)
plt.xlabel("Day2-The number of bee visits")
plt.ylabel("Count")
plt.grid(axis = "y",zorder = 0)
plt.hist(Visit_D2 , bins = 20, density = False, range = (0, 20),zorder = 10,color="lightseagreen",label="Day 2")
plt.legend()

plt.subplot(2,3,3)
plt.xlabel("Day3-The number of bee visits")
plt.ylabel("Count")
plt.grid(axis = "y",zorder = 0)
plt.hist(Visit_D3 , bins = 20, density = False, range = (0, 20),zorder = 10,color="lightseagreen",label="Day 3")
plt.legend()
```

```
plt.subplot(2,3,4)
plt.xlabel("Day4-The number of bee visits")
plt.ylabel("Count")
plt.grid(axis = "y",zorder = 0)
plt.hist(Visit_D4 , bins = 20, density = False, range = (0, 20),zorder = 10,color="lightseagreen",label="Day 4")
plt.legend()


plt.subplot(2,3,5)
plt.xlabel("Day5-The number of bee visits")
plt.ylabel("Count")
plt.grid(axis = "y",zorder = 0)
plt.hist(Visit_D5 , bins = 20, density = False, range = (0, 20),zorder = 10,color="lightseagreen",label="Day 5")
plt.legend()


plt.tight_layout()
plt.show()


# Figure 2 蜜蜂放置的花粉数量分布
plt.subplots(figsize=(9, 6),dpi= 300)
plt.subplot(2,3,1)
plt.xlabel("Day1-Number of deposited pollen")
plt.ylabel("Count")
plt.grid(axis = "y",zorder = 0)
plt.hist(All_Pollen_D1 , bins = 20, density = False, range = (0, 500),color="tomato",zorder = 10,label="Day 1")
plt.legend()
```

```
plt.subplot(2,3,2)

plt.xlabel("Day2-Number of deposited pollen")

plt.ylabel("Count")

plt.grid(axis = "y",zorder = 0)

plt.hist(All_Pollen_D2 , bins = 20, density = False, range = (0, 500),color="tomato",zorder = 10,label="Day 2")

plt.legend()


plt.subplot(2,3,3)

plt.xlabel("Day3-Number of deposited pollen")

plt.ylabel("Count")

plt.grid(axis = "y",zorder = 0)

plt.hist(All_Pollen_D3 , bins = 20, density = False, range = (0, 500),color="tomato",zorder = 10,label="Day 3")

plt.legend()


plt.subplot(2,3,4)

plt.xlabel("Day4-Number of deposited pollen")

plt.ylabel("Count")

plt.grid(axis = "y",zorder = 0)

plt.hist(All_Pollen_D4 , bins = 20, density = False, range = (0, 500),color="tomato",zorder = 10,label="Day 4")

plt.legend()


plt.subplot(2,3,5)

plt.xlabel("Day5-Number of deposited pollen")

plt.ylabel("Count")

plt.grid(axis = "y",zorder = 0)

plt.hist(All_Pollen_D5 , bins = 20, density = False, range = (0, 500),color="tomato",zorder = 10,label="Day 5")
```

```
plt.legend()

plt.tight_layout()
plt.show()

# Figure 3  成功受孕的花粉数量分布
plt.subplots(figsize=(9, 6),dpi= 300)
plt.subplot(2,3,1)
plt.xlabel("Day1-Fertilized pollen Number")
plt.ylabel("Count")
plt.grid(axis = "y",zorder = 0)
plt.hist(F_Pollen_D1 , bins = 18, density = False, range = (0, 300),color="cornflowerblue",zorder = 10)

plt.subplot(2,3,2)
plt.xlabel("Day2-Fertilized pollen Number")
plt.ylabel("Count")
plt.grid(axis = "y",zorder = 0)
plt.hist(F_Pollen_D2 , bins = 18, density = False, range = (0, 300),color="cornflowerblue",zorder = 10)

plt.subplot(2,3,3)
plt.xlabel("Day3-Fertilized pollen Number")
plt.ylabel("Count")
plt.grid(axis = "y",zorder = 0)
plt.hist(F_Pollen_D3 , bins = 18, density = False, range = (0, 300),color="cornflowerblue",zorder = 10)

plt.subplot(2,3,4)
plt.xlabel("Day4-Fertilized pollen Number")
```

```
plt.ylabel("Count")
plt.grid(axis = "y",zorder = 0)
plt.hist(F_Pollen_D4 , bins = 18, density = False, range = (0, 300),color="cornflowerblue",zorder = 10)

plt.subplot(2,3,5)
plt.xlabel("Day5-Fertilized pollen Number")
plt.ylabel("Count")
plt.grid(axis = "y",zorder = 0)
plt.hist(F_Pollen_D5 , bins = 18, density = False, range = (0, 300),color="cornflowerblue",zorder = 10)

plt.subplot(2,3,6)
plt.xlabel("Total number in 5 days")
plt.ylabel("Count")
plt.grid(axis = "y",zorder = 0)
plt.hist(F_Pollen_All , bins = 18, density = False, range = (0, 600),color="royalblue",zorder = 10)
plt.tight_layout()
plt.show()

# Figure 4 weight distribution
plt.figure(dpi = 150)
plt.hist(weight, bins = 20, density = False, range = (0, 35))
plt.xlabel("fruit weight (g)")
plt.show()

print(sum(weight)/len(weight))

total = 0
```

```
for i in range(flower_number):
    if weight[i] >= 10:
        total = total + 1


# Figure 5
plt.figure(dpi = 300)
plt.hist([weight,weight_r], bins = 20, density = True, range = (0, 35),label=['Simulated berry data', 'Empirical data'],color = ["lightseagreen","tomato"],zorder = 10)
plt.xlabel("fruit weight (g)")
plt.ylabel("Frequency")
plt.grid(axis = "y",zorder = 0)
plt.legend()
plt.savefig('F5.svg', dpi = 300, format='svg')
plt.show()


# Figure 6
u_6 = 14.30
sigma_6 = 4.42
x_6 = np.arange(0, 30, 0.1)
y_6 = np.exp(-((x_6 - u_6)**2)/(2*sigma_6**2)) / (sigma_6 * np.sqrt(2*np.pi))


plt.figure(dpi = 150)
label_6 = "λ = " + str(rou)
plt.hist(weight, bins = 20, density = True, range = (0, 35),color = "slateblue",zorder = 10, label = label_6)
plt.plot(x_6, y_6, 'k--', linewidth = 2 ,zorder = 20)
plt.xlabel("fruit weight (g)")
plt.ylabel("Frequency")
plt.grid(axis = "y",zorder = 0)
plt.legend()
```

```
plt.show()
```
