# Supplementary material for: Why does strawberry fruit weight distribution show positive skewness? A simulation model reveals the underlying processes of fruit production
Source: Front Plant Sci. 2023 Dec 14;14:1255724. doi: 10.3389/fpls.2023.1255724 (PMC11790150; doi:10.3389/fpls.2023.1255724)
Supplement: Supplementary file 2 [file DataSheet_2.docx]

**Part 1. The Validation for “2.1 Bee pollination process”**


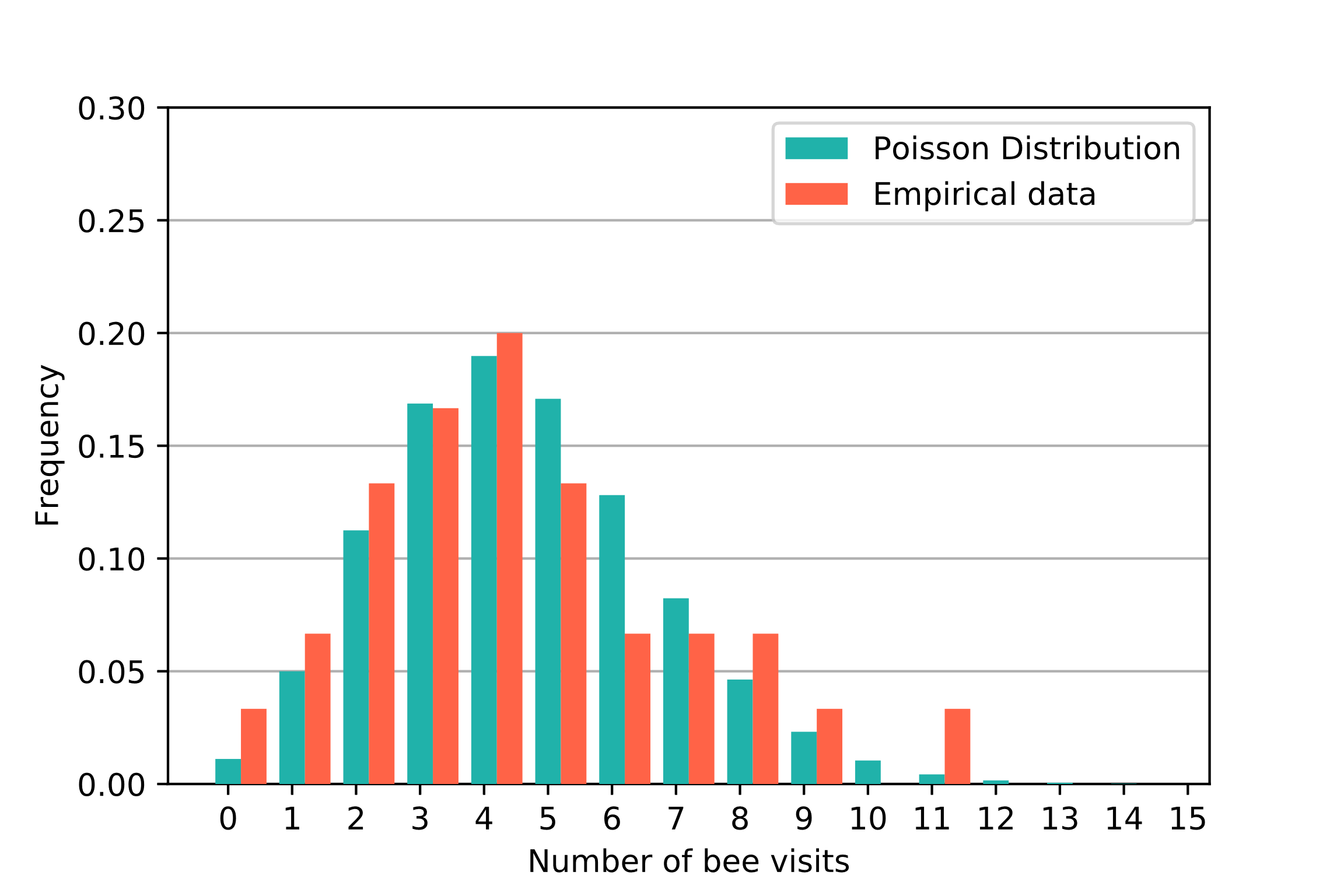


**FigS1**. Graphical representation of the distribution for the frequency of bee visits to strawberry flowers in a greenhouse.

In our preliminary research, we randomly selected 30 strawberry flowers from a greenhouse and meticulously recorded the number of bee visits to these flowers within a day. The resulting probability distribution graph is presented in Figure S1 as the red histogram, while the green histogram represents the probability distribution of a Poisson distribution with a standard parameter $\lambda$ of 4.5. The orange histogram represents the probability distribution of empirical data of those 30 strawberry flowers selected.

It is evident that the two distributions bear a striking resemblance. We conducted a one-sample Kolmogorov-Smirnov test to assess the fit of a Poisson distribution to the empirical data consisting of these 30 strawberries. The test yielded a p-value > 0.05. This outcome suggests that the Poisson distribution adequately captures the distribution characteristics of the daily number of bee visits to individual strawberry flower within the greenhouse setting.

**Part 2. The Validation for “2.2 Pollen fertilization process”**

The line chart depicting $P_{n}^{r}$ in equation (3) is shown in Figure S2.


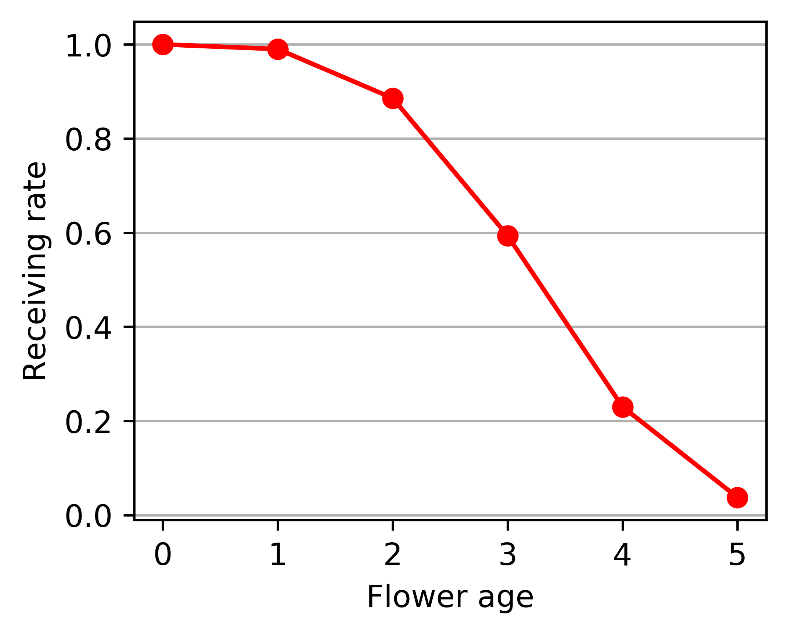


**Fig2**. Stigma receptivity of flowers at different developmental ages

In Figure S3(a), the correlation between the number of achenes and the weight of 50 *Beni hoppe* strawberry fruits ($w_{1},w_{2}\ldots w_{50}$) is depicted. The 50 strawberries were randomly harvested from an actual greenhouse, making them a representative sample of real-world data. The red dots represent the number and weight information of each fruit.

By calculating the residuals between the actual strawberry data and the fitting results, and then utilizing the one-sample Kolmogorov-Smirnov test, we obtained the result that p-value > 0.05 (p-value = 0.2), indicating that $Bias$ basically follows a normal distribution.


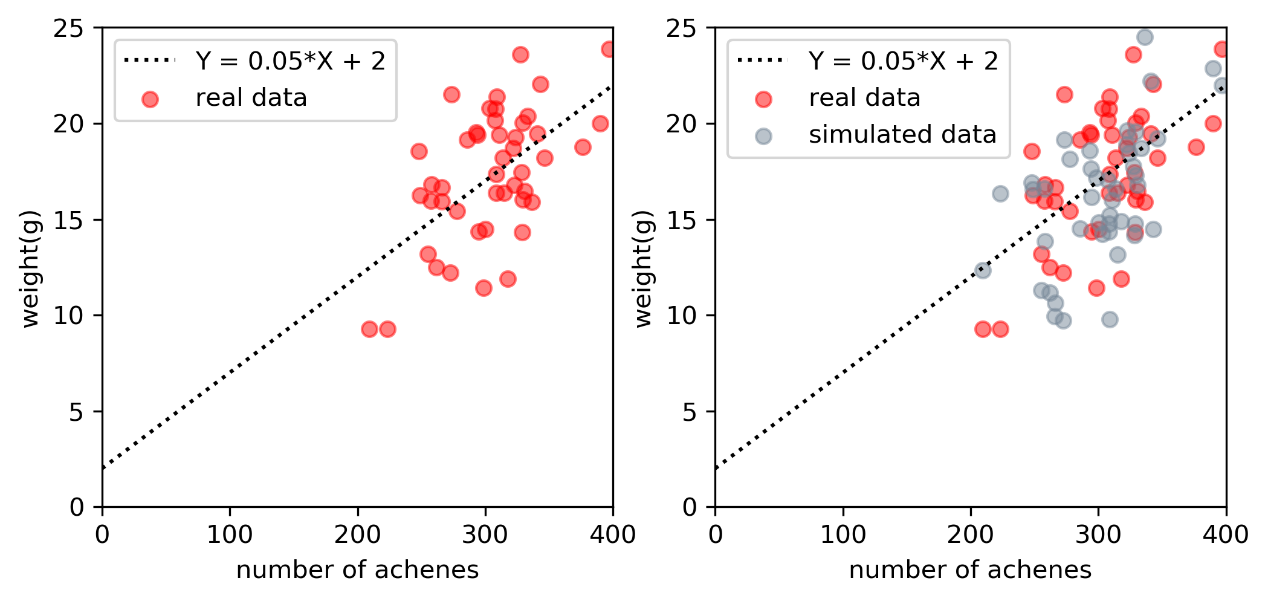


(a) (b)

**Fig.S3** (a) Empirical data of 50 strawberries is represented by red dots, and the fitting curve is represented by a dashed line; (b) The simulated strawberry information is represented by gray dots.

## Part 3. Fruit growth process

The calculation indicated that the unbiased estimate of $\sigma^{2}$ is 5.6 in equation (7). Therefore, in the simulation part of this study, the residual between fruit weight and linear fitted result follows $Bias\sim N(0,5.6)$. In the simulation experiment, the data is input into formula (7) for actual calculation. The gray dots in Figure S3(b) represent the relationship between the number of achenes and fruit weight $W$ in the simulation results. We employed the Kolmogorov-Smirnov test to evaluate the significant difference between the simulated data and the empirical data. The test results indicated a p-value exceeding 0.05 which revealed that there is no significant difference between the two sets of data. From the experimental results, it can be concluded that the simulated data can accurately represent the range of residual fluctuations.

To estimate the value of the standard deviation $\sigma$ for the $Bias$ distribution, the fruit weights ($w_{1},w_{2}\ldots w_{50}$) of the 50 real strawberries collected in the greenhouse can be utilized. The unbiased estimate of $\sigma^{2}$ can be calculated using following equation.

$$\sigma^{2}=\frac{1}{n-1}\sum_{i=1}^{n} \left( w_{i} \right)$$
